# Mechanisms of Atrial Fibrillation: How Our Knowledge Affects Clinical Practice

**DOI:** 10.3390/life13061260

**Published:** 2023-05-25

**Authors:** Georgios Leventopoulos, Rafail Koros, Christoforos Travlos, Angelos Perperis, Panagiotis Chronopoulos, Evropi Tsoni, Eleni-Evangelia Koufou, Athanasios Papageorgiou, Anastasios Apostolos, Panagiotis Kaouris, Periklis Davlouros, Grigorios Tsigkas

**Affiliations:** Cardiology Department, University Hospital of Patras, 26504 Patras, Greece; korosraf@hotmail.com (R.K.); christof1999@gmail.com (C.T.); angelosperperis@msn.com (A.P.); takaroschr@gmail.com (P.C.); eriplatani@hotmail.com (E.T.); elenikoufou17@gmail.com (E.-E.K.); thanasispapag95@gmail.com (A.P.); fromfleet7@gmail.com (A.A.); kaourispk@gmail.com (P.K.); pdav@upatras.gr (P.D.); gregtsig@hotmail.com (G.T.)

**Keywords:** atrial fibrillation, fibrosis, triggers, inflammation oxidative stress

## Abstract

Atrial fibrillation (AF) is a very common arrhythmia that mainly affects older individuals. The mechanism of atrial fibrillation is complex and is related to the pathogenesis of trigger activation and the perpetuation of arrhythmia. The pulmonary veins in the left atrium arei confirm that onfirm the most common triggers due to their distinct anatomical and electrophysiological properties. As a result, their electrical isolation by ablation is the cornerstone of invasive AF treatment. Multiple factors and comorbidities affect the atrial tissue and lead to myocardial stretch. Several neurohormonal and structural changes occur, leading to inflammation and oxidative stress and, consequently, a fibrotic substrate created by myofibroblasts, which encourages AF perpetuation. Several mechanisms are implemented into daily clinical practice in both interventions in and the medical treatment of atrial fibrillation.

## 1. Introduction

Atrial fibrillation (AF) is the most frequent cardiac arrhythmia and is linked with remarkable mortality and morbidity, which are caused by thromboembolism, heart failure, and impaired cognitive function [1]. The prevalence of AF was estimated in the first years of the 21st century to lie within a range between 0.5% and 1% in the general population, but currently it seems to range between 2% and 4% in developed countries [2]. A threefold increase in its prevalence has been observed over the last 50 years [3]. The risk of AF increases with each decade and exceeds 20% by the age of 80 years [4].

The main ECG characteristics of AF include a lack of discrete P waves and the rapid appearance of fibrillatory (or f) waves that vary in amplitude, morphology, and rate. The QRS complexes demonstrate irregular R–R intervals. The atrial rate may be between 350 and 600 beats per minute (bpm) or unmeasurable.

Fibrillatory waves are usually best seen in the inferior leads and in V1. They may be identified between QRS complexes and are sometimes visible superimposed on the ST segment and T waves [5]. The recording of AF in a single-lead ECG for ≥30 s or in a 12-lead ECG results in the explicit diagnosis of AF [6]. Furthermore, AF is classified as follows: (a) paroxysmal, if it terminates spontaneously or with either (i) electric or (ii) pharmacological cardioversion within a week of onset; (b) persistent, if it is ceaselessly sustained beyond a week, including episodes terminated by intervention after ≥7 days; (c) long-standing and persistent, if it is continuous for over a year and a rhythm-control strategy is adopted; or (d) permanent if it is accepted by the patient and physician, and no other medical intervention with the aim of restoring/maintaining rhythm is conducted [7]. However, this classification does not refer to the underlying atrial substrate, which determines long-term sinus-rhythm maintenance, in cases in which a rhythm-control strategy is selected. The disorganized atrial contractions of AF lead to a 20–30% reduction in stroke volume and cardiac output in healthy individuals. A considerable decline in output occurs in cases of diastolic dysfunction in which atrial contraction contributes more than normal to left ventricular diastolic filling [8].

**Figure 1:**
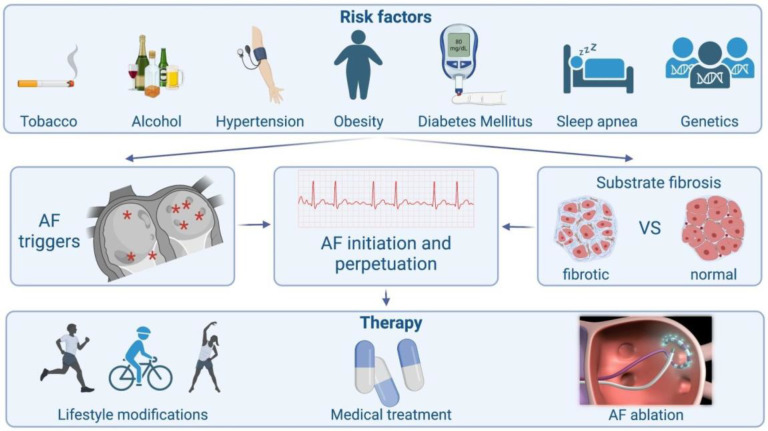
Schematic presentation of atrial fibrillation from causes to treatment.

The basic pathophysiological pathways leading to AF’s onset and perpetuation involve triggers, abnormal atrial substrates, neurohormonal hyperactivation and, finally, a genetic predisposition (Figure 1).

Our aim in this review is to delineate the mechanisms of AF based on the cellular and electrophysiologic substrate and describe how these proposed mechanisms are implemented into daily clinical practice in both interventions in and the medical treatment of AF.

## 2. Triggers

Typically, premature atrial beats are the main triggers that convert sinus rhythm to AF. According to Haïssaguerre et al., the vast majority of these ectopic foci inside the atria originate in the pulmonary veins (PVs). The myocardial sleeves within the PVs appear to have specific properties that are both similar to and different from those of the rest of the atrial myocytes in terms of cellular electrophysiology, anatomical characteristics, and myofiber structure and orientation. The main aim of AF catheter ablation is the electrical isolation of the PVs from the rest of the atrium. This therapeutic strategy constitutes the cornerstone of AF catheter ablation [9]. Arrhythmogenesis has a predilection toward PV cardiomyocytes due to their action-potential characteristics, which renders them more susceptible to enhanced normal automaticity and triggered activity.

It is accepted that the acceleration of phase 4 depolarization results in enhanced normal automaticity, reaching an earlier threshold and an elevated automatic rate. On the other hand, delayed after-depolarization is the outcome of intracellular Ca^+2^ overload. In this process, he calcium-overloaded sarcoplasmic reticulum excretes Ca^+2^ in the diastole, activates Ca^+2^-dependent depolarizing currents (such as the Na^+^/Ca^+2^ exchange current) and, subsequently, produces a transient inward current that provokes membrane depolarization. The delayed after-depolarization reaches its threshold and a triggered ectopic action potential ensues. Early after-depolarizations originate from disproportionate action-potential prolongation caused by (a) the loss of repolarizing K^+^ currents, (b) excessive late components of Na^+^ currents and (c) the reactivation of plateau Ca^+2^ currents, which produce secondary arrhythmic depolarizations [10]. It is apparent that PV cells fulfill these criteria. The PVs have a depolarized resting membrane potential—which facilitates enhanced normal automaticity. Furthermore, PVs also provide an action potential with a lower amplitude, a shorter duration, and a smaller maximum phase 0 upstroke velocity. Slow and rapid delayed K^+^ rectifier currents are augmented in the PVs, whereas transient outward K^+^ currents and L-type Ca^2+^ currents are attenuated. Furthermore, animal studies have demonstrated that the diminished activity of the IK1 current facilitates trigger activity during the late phase of depolarization based on an afterdepolarization effect [11].

**Figure 2:**
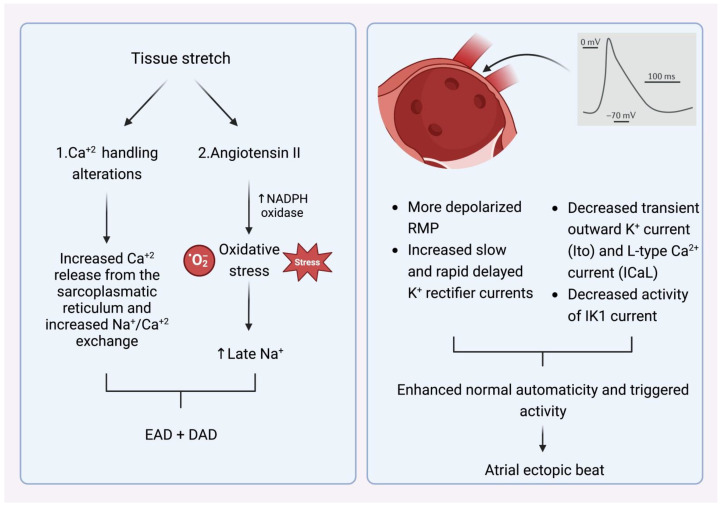
(**Left**) Mechanism that explains how tissue stretch promotes atrial premature beats, mainly through afterdepolarization. (**Right**) Specific electrophysiologic properties of the pulmonary veins render them more likely to be the origin of atrial ectopic beats. *NADPH oxidase* (nicotinamide adenine dinucleotide phosphate oxidase) EAD: early afterdepolarization. DAD: delayed afterdepolarization. RMP: resting membrane potential.

What is, however, the crucial stimulus that provokes the premature atrial beats in the pulmonary veins? The main AF comorbidities, such as hypertension, ischemic cardiomyopathy, diabetes mellitus, congestive heart failure, and advanced age alter left ventricular elastic properties and subsequently increase left atrial pressure [12]. The most common last step in this pathophysiologic cascade is the development of tissue stretch, which can also induce afterdepolarization and, thus, ectopic activity. This is facilitated by alterations in Ca^+2^ handling induced by increased Ca^+2^ excretion from the sacroplasmatic reticulum and enhanced Na^+^/Ca^+2^ exchange, particularly in the setting of b-adrenergic stimulation. Additionally, angiotensin II contributes similarly to Ca^+2^-handling changes by activating nicotinamide adenine dinucleotide phosphate (NADPH) oxidase, which is a significant downstream effector in cellular oxidative stress. It is known that NADPH oxidase generates reactive oxygen species (ROS). In turn, ROS products induce early afterdepolarization (EAD) and delayed afterdepolarization (DAD) as a trigger activity through the enhancement of late Na^+^ currents. Angiotensin II and ROS enhance abnormal Ca^+2^ handling and Ca^+2^ overload, which cause increased atrial contractility and upregulate Ca^+2^-dependent signaling. The subsequent alteration of the intracellular calcium balance encourages early after-depolarization. The Ca^+2^-induced activation of the nuclear factor of activated T cells (NFAT) suppresses the L-type Ca^+2^-current function, decreases action-potential duration (APD), and facilitates AF-induced electrical remodeling [13] (Figure 2).

**Figure 3:**
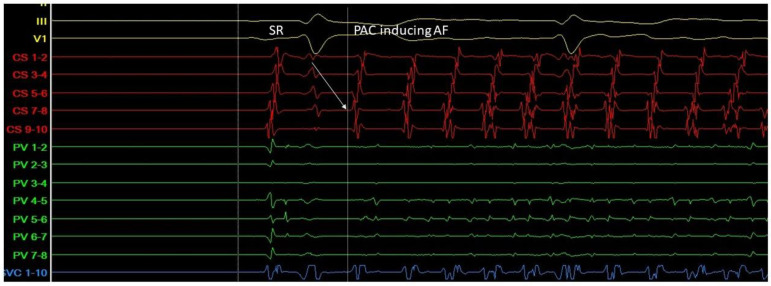
A. Atrial ectopic beat originates from the proximal aspect of the coronary sinus (arrow at the CS 7-8 electrogram)—a non PV trigger—and induces atrial fibrillation (G. Leventopoulos’ archive). PV: pulmonary vein. CS: coronary sinus.

**Figure 4:**
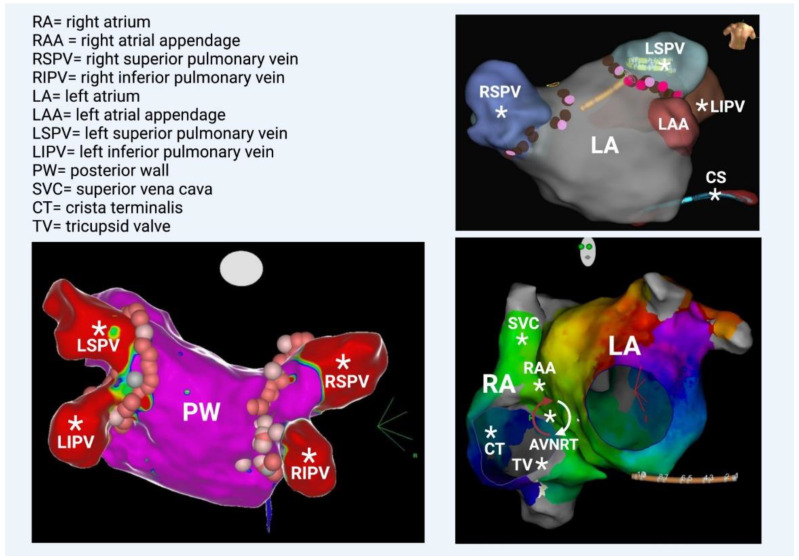
(**Bottom left**) Left atrium—posterior view. All 4 veins and the posterior wall are illustrated; they share the same embryologic origin. (**Top right**) Left atrium—anterior view. Note the left atrial appendage and the coronary sinus (catheter placed inside), which are rarely additional PV triggers. (**Bottom right**) Left anterior oblique view. The anatomical correlation between right and left atrium is depicted. Geometry of both atria was created in a patient with perimitral flutter, in this particular case. Generally, several additional PV triggers are also located in the right atrium, such as the superior vena cava, the right atrial appendage, the crista terminalis, and the tricuspid valve. Occasionally, AF involves the degeneration of AVNRT (atrioventricular nodal reciprocating tachycardia). * Potential anatomical origins of atrial ectopic beat.

Triggers may originate from different regions (non-PV triggers), including the superior vena cava, crista terminalis, coronary sinus, left atrial appendage, left atrial posterior free wall, and ligament of Marshall [14]. Specific mapping protocols and isoprenaline have been used to address the significance of inducing, locating, and eliminating such non-PV triggers as a means to achieving better results in AF ablation in comparison to empirical PVI. However, this therapeutic strategy has not been established as a common practice [15] (Figure 3 and Figure 4).

Recently, the role of posterior-LA-wall isolation has emerged. The posterior LA wall should be viewed as an extension of the PVs with clear arrhythmogenic potential, considering its embryological, electrophysiological, and anatomical properties. During human embryogenesis, PVs initially have no link with the fetal heart. At later stages, they converge and form a common PV (at about the fifth or sixth month in utero), which becomes incorporated into the left atrium (LA) by connecting to the embryological LA on the posterior aspect. Subsequently, a smooth posterior LA is formed [16]. It is known that the posterior wall serves as an area in which AF triggers are located. Different stimulation protocols using atrial burst with or without isoprenaline have shown ectopic atrial beats that originate from the posterior wall and lead to AF initiation. Thus, there is a long-standing debate over whether empirical posterior wall isolation (PWI) on top of PVI would have an additional beneficial effect on arrhythmia-free patients’ outcomes. This proposal has advocated most strongly for patients with persistent AF. Most often, posterior wall isolation is achieved by creating a roof and a posterior line through continuous RF lesions. A multipolar catheter is used for the verification of electrical isolation. The ablation therapy aims to electrically isolate the posterior wall [15]. However, in the most recent randomized CAPLA study, patients with persistent AF underwent catheter ablation for the first time. It was proven that the patients who were treated with PWI in combination with PVI did not show significant improvements in freedom from atrial arrhythmia at one year compared with those treated with PVI alone. In light of the outcomes of this trial, the empirical inclusion of PWI for the ablation of persistent AF was not supported [17]. Lastly, regarding the additional PV triggers, it should be underlined that in some cases, AF is related to AVNRT or AVRT, and this scenario should be given particular consideration in young individuals in whom the “traditional” AF risk factors are absent. The atrial activity in these well-defined reentrant circuits can be easily degenerated, transforming regular QRS tachycardia (AVNRT/AVRT) into chaotic atrial propagation, which is indicative of atrial fibrillation [18].

## 3. AF Perpetuation

Although triggers are required for AF initiation, a vulnerable atrial substrate is equally important. Structural and electrophysiological atrial abnormalities assist AF perpetuation. Reentry constitutes a fundamental mechanism in the maintenance of AF in two possible ways: (a) reentrant rotors or (b) multiple independent wavelets. Multiple wavelets are multiple, simultaneous re-entrant circuits within the atria [19]. The electric dissociation of epicardial and endocardial layers has also been considered to promote reentry and contribute to the perpetuation of AF [20]. Rotors are self-sustaining rotational circuits with a spiral-wave morphology. In the propagation of models shaped in this way, the greatest curvature—at the inner part of the spiral—has the slowest propagation velocity, creating an area of functional conductional blockage at the center of the rotor as the wavelet constantly confronts an unexcitable core. However, this model proposes that the rotor is not stationary, but instead rotates around the atrial tissue. By contrast, the less accepted theory of the “leading circle” includes a stationary unexcited core. Stable rotors can anchor at certain sites—often around PVs and in areas of heterogeneous atrial tissue—forming wavefronts that spread away from the center of the rotor and then fragment, inducing chaotic and fibrillatory activity within the rest of the atrium [21].

Based on the considerations above, FIRMap (focal impulse and rotor modulation) uses phase mapping to identify the locations of up to three rotors or focal sources. This is achieved by a 64-lead basket catheter, which provides endocardial unipolar electrograms. The FIRM maps of AF reveal electrical rotors defined as sequential clockwise or counterclockwise activation contours around a center of rotation emitting outward to sustain AF activation, or focal impulses defined by centrifugal activation contours from an origin. The CONFIRM study revealed for the first time that AF may be perpetuated by localized sources in the form of electrical rotors and focal impulses. The use of FIRM ablation at patient-specific AF-sustaining sources terminated or consistently prolonged the cycle length in persistent or paroxysmal AF compared to conventional ablation in 86% of patients, and substantially increased long-term AF elimination using extremely careful monitoring compared to conventional AF ablation alone. Consistently, favorable outcomes were also reported by three other small single-center studies [22,23,24]. However, multicenter trials, recent systematic reviews, and meta-analyses of AF-rotor and -driver ablation have shown high variability and discrepancy in success rates, which do not seem to be superior to conventional pulmonary vein ablation alone. A similar approach was attempted with a 252-electrode body-surface vest in order to obtain electrocardiographic imaging (ECGi) and to demonstrate rotors that—as opposed to the previous FIRM method—were transient for several cycles only [25].

Structural and electrical remodeling are necessary for the creation of the appropriate substrate and the initiation of reentrant rotors or fragmented wavelets with AF perpetuation as the final result. Ausma et al. noted that rapid pacing induced significant cellular structure changes, such as the formation of enlarged and disordered fibers, enlarged nuclei, giant mitochondria, and a dilated sarcoplasmic reticulum [26]. Similarly, abnormal histological findings were uniformly found in multiple atrial biopsy specimens in all patients with lone AF by Frustaci et al. [27]. Structural remodeling is characterized by changes in tissue properties and cellular ultrastructure, leading to atrial dilatation, which is the most common echocardiographic finding in patients with AF. Atrial fibrosis has a major role in structural remodeling and it is caused by the deposition of extracellular matrix proteins in the myocardial interstitial tissue. These changes predispose patients to defects in conduction, predominantly contributing to reentry and rotor formation. Atrial fibrosis is caused by the transformation of fibroblasts into myofibroblasts after the activation of transcription factors, and has classically been ascribed to ageing, comorbidities, and cardiovascular risk factors [28].

Many pathways are responsible on a molecular level for fibrosis formation. The renin–angiotensin–aldosterone system is involved in myocardial fibrosis, which is induced by medical conditions such as heart failure, cardiomyopathies, hypertension, and ischemic heart disease. Angiotensin II (Ang II) activates the production of (a) transforming growth factor-β (TGF-β) and (b) extracellular matrix (ECM) proteins, which form AF-promoting fibrous tissue.

The TGF-β1 is a major established profibrotic signaling molecule and a positive regulator of cardiac fibrosis. Its specific overexpression in the heart leads to atrial fibrosis and, in turn, increased susceptibility to AF. Exposure to Ang II or TGF-1 influences cardiac fibroblast function. Excessive fibroblast proliferation leads to the increased synthesis, secretion, and deposition of extracellular-matrix protein and subsequent fibrosis in the interstitial and perivascular space [29]. Both AngII production and AT1-receptor expression are increased during remodeling in fibroblasts in vivo. Increases in Ang II and activated TGF-1 concentrations reciprocally enhance each other’s production, the transformation of fibroblasts into collagen-secreting myofibroblasts as the final step in this process. As a result, collagen deposition and fibrosis facilitate AF perpetuation. Moreover, mechanical stretch induces collagen synthesis, along with increased Ang II and TGF-1 expression in cardiac fibroblasts. Thus, chronic atrial dilation may contribute to structural remodeling and the maintenance of AF [30,31].

Apart from myofibroblast proliferation, ion-channel remodeling may also contribute to AF pathogenesis. Cardiac fibroblast proliferation and differentiation is regulated by Ca^+2^-permeable transient receptor potential (TRP) canonical-3 (TRPC3) channels. It was proven that TRPC3 expression is increased in the atria of AF patients. In turn, AF enhances TRPC3-channel expression by causing the NFAT-mediated downregulation of microRNA-26 and results in the TRPC3-dependent enhancement of fibroblast proliferation and differentiation. In vivo, TRPC3 blockade was demonstrated to prevent AF-substrate development in a dog model of electrically maintained AF. These findings suggest the role of TRPC3 in AF, which involves its mediation of fibroblast pathophysiology, and it can be considered a novel potential therapeutic target [32].

Reactive oxygen species (ROS) refer to low-weight molecules, which constitute derivatives through oxygen metabolism. In heart-failure patients, ROS results in increased calmodulin-dependent protein kinase II (CAMKII) oxidation in the left posterior atrium, which encourages conduction delay and increased conduction heterogeneity, creating appropriate substrates for reentry circuits. Furthermore, superoxide and H_2_O_2_ products promote myocyte-apoptosis fibrosis and inflammation [33].

ROS production can be estimated and measured by stable markers in the circulation, including markers of lipid peroxidation (isoprostanes), malondialdehyde, oxidized phospholipids, myeloperoxidase, nitrotyrosine, and aminothiol compounds. A 10% increase in glutathione (Eh GSH) levels was linked with a 30% increase in AF incidence. Several therapeutic approaches to AF management have been designed. The use of oxygen-radical scavengers for ROS suppression, such as vitamin C, vitamin E, ebselen, tempol and N-acetyl-cysteine, have been considered. In this direction, therapeutic agents for use against the sources of ROS generation and their key signaling mediators have also been tested [34,35].

Several immune cellular mediators are involved in the fibrotic process that creates the appropriate substrate for AF sustainability. Following myocardial injury, monocytes initiate their differentiation into inflammatory macrophages, which play a significant role in the initiation and progression of fibrotic responses. Resident macrophages contribute to inflammatory myocardial microenviroments by recruiting other inflammatory leukocytes and by secreting metalloproteinase (MMP) 2, MMP 9, and inflammatory markers (IL-6), which regulate fibroblast function [36].

IN addition to macrophages, T-cell infiltration of and recruitment to injured areas as an outcome of cytokine release play a crucial role in atrial fibrosis. For instance, Th1 cells demonstrate antifibrotic action by releasing IFN-γ, inhibiting the TGF-β pro-fibrotic activity and suppressing collagen I and its expression. On the other hand, Th2 cells release pro-fibrotic mediators, such as IL4 and IL13, stimulating collagen synthesis. Furthermore, CD8+ T-cells are involved in the activation of the removal of necrotic debris and collagen-scar formation mediated by macrophages [37].

Furthermore, mast cells have been proven to participate in atrial fibrosis. They produce significant fibrosis mediators, including proteases (i.e., tryptase and chymase) and growth factors (e.g., TGF-β1, TNF, and IL-1) [38]. Tryptase and chymase (a significant protease in the conversion Ang I into Ang II) are major mediators of fibroblast physiology and exert modulatory effects on the atrial substrate and the increase in fibroblast proliferation. Consequently, collagen synthesis and myocardial fibrosis are induced [39,40].

Apart from structural remodeling, other changes in electrophysiological atrial properties, referred to as electrical remodeling, are among the most significant driving factors in AF perpetuation. Electrical remodeling consists of (a) the suppression of the Ca^+2^ current, which shortens the refractory period, (b) the enhancement of outward K^+^ currents, leading to the accelerated repolarization and hyperpolarization of atrial cells, and (c) the modified expression and localization of connexins, resulting in conduction abnormalities. The efficacy of antiarrhythmic drugs in AF, such as class I agents (which block Na^+^), class III agents (which block K^+^), or class IV agents (non-dihydropyridine Ca^++^ blockers), is explained by these mechanisms [41]. Electric remodeling is characterized by AF-induced shortening in action-potential duration (APD) and increases in delayed afterdepolarization (DAD) risk. By shortening the APD, atrial reentry rotors appear more stable, increasing AF vulnerability and sustainability. In addition, alterations in Ca^+2^ handling encourage diastolic Ca^+2^ release and ectopic activity. Electrical remodeling can explain why AF can recur early after cardioversion, progress from paroxysmal to more persistent forms, or develop drug resistance [42].

Connexin-40 and connexin-43 are gap-junction proteins, responsible for cell-to-cell electrical conduction [43]. Their expression is changed in AF patients, potentially assisting re-entry-encouraging conduction abnormalities. There is controversy among studies over whether reduced or increased connexin-40 expression at the transverse cell membrane encourages heterogeneous conduction. Abnormal connexin amounts, along with fibrosis deposits, account for zig-zag conduction delays, which encourage the induction of reentry arrhythmias through regions of slowing conduction velocity and unidirectional block. These are the fundamental principles of reeentry circuits.

**Figure 5:**
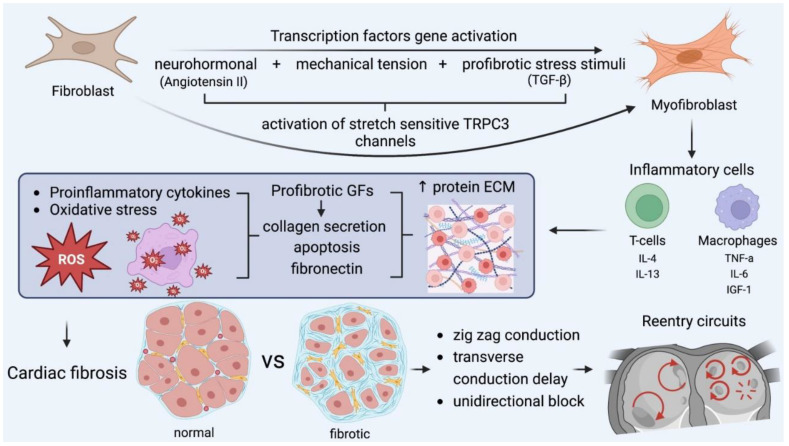
The key role of myofibroblasts in fibrosis formation and the main pathophysiologic cascades. Mechanical stress accounts for myocardial injury, oxidative stress, and neurohormonal activation, which transform fibroblasts into myofibroblasts, which, along with other inflammatory cells, encourage fibrotic substrates. In this microenviroment, all the required conditions for the formation and perpetuation of reentry circuits are fulfilled and further enhanced. GF: growth factor, IL: interleukin, TGF: transforming growth factor, TNF, tumor necrosis factor. IGF: insulin growth factor.

It is apparent that all the above-mentioned pathways affect AF perpetuation and lead to the established perception that *AF begets AF* [44] (Figure 5).

From this perspective, the extent of atrial fibrosis is an adverse prognostic sign in AF treatment, as shown in the DECAAF study, which used late gadolinium enhanced (LGE) MRI to provide a noninvasive means of estimating atrial fibrosis [45]. Based on this, the DECAAF II randomized trial supported the hypothesis that the addition of image-guided fibrosis ablation to conventional PVI results in higher procedural success rates in patients with persistent AF due to fibrotic-tissue debulking. Trial findings revealed that baseline fibrosis predicted AF-ablation outcomes, especially when fibrosis was present at increased levels, but no statistically significant differences were observed in the primary endpoint (time to first arrhythmia recurrence) between groups in the total study population. The subgroup analyses revealed a tendency towards a lower rate of atrial arrhythmia recurrence in the intervention group for patients with a lower extent of fibrosis at baseline. This is another example of how our understanding of the pathophysiology of fibrosis still cannot be translated into clinical benefits by current therapeutic tools [46].

## 4. The Role of the Autonomous Nervous System

Furthermore, it should be emphasized that the heart is richly innervated by the autonomic nerves, forming clusters called ganglia either outside (extrinsic) or inside of the organ (intrinsic). Preganglionic cardiac parasympathetic axons arise from various nuclei in the medulla, run in the cardiac branches of the vagus nerve, and synapse in cardiac plexuses and intrinsic ganglia, giving rise to postgangionic parasympathetic fibers. The extrinsic sympathetic nerves arise from the paravertebral ganglia, including the superior cervical ganglion, the middle cervical ganglion, the thoracic ganglia, and the cervicothoracic (stellate) ganglion. Abnormal autonomic innervation and AF are linked. In addition to atrial sympathetic hyperinnervation, diseases also cause the remodeling of extracardiac nerve structures in both experimental animals and humans [47]. Both b-adrenergic and cholinergic stimulations have been used for decades to provoke AF in experimental studies. Similarly, human and animal studies have shown alterations in sympatho-vagal balance during AF onset. Concisely, rapid rates of AF were found to lead to a heterogeneous increase in atrial sympathetic innervation. Increased sympathetic/parasympathetic fibers and enlarged cardiac ganglia have been observed in the posterior left atria and pulmonary veins of heart-failure patients, encouraging AF maintenance. Augmented parasympathetic tone with enhanced baroreceptor responsiveness and increased cardiomyocyte sensitivity to cholinergic stimulation were observed with atrial dilatation and fibrosis following chronic endurance training, leading to AF susceptibility [48].

## 5. Novel Concepts in AF Mechanisms—The Role of Pericardial Adipose Tissue

Additionally, a novel emerging concept in AF pathogenesis is the role of pericardial adipose tissue. It comprises the pericardial fat situated outside the visceral pericardium and the epicardial adipose tissue located between the visceral pericardium and the epicardium. Intra-myocardial fat deposits that are dedicated to triglyceride storage in the myocardium coexist. Pericardial and epicardial fat differ embryologically, but both evolve from brown adipose tissue, as intra-abdominal visceral fat. Some white adipose tissue can be detected in the atrioventricular and inter-ventricular grooves of the adult heart. Epicardial adipose tissue and the neighboring myocardium are considered to be connected, as there are no distinct intervening fascia between the two tissues, in contrast to the anatomical separation of the pericardial fat from the myocardium by the visceral pericardium. There are several mechanisms associated with pericardial fat and AF. Firstly, pericardial fat is considered to increase left ventricular mass and, consequently, impair diastolic function. In addition, the fatty infiltration of the ventricular myocardium and atrial septum leads to electromechanical alterations in the atrial myocardium. Pericardial fat has paracrine properties and stimulates the release of inflammatory mediators, including tumor necrosis factor-α and interleukin-6, which encourage local inflammation and, subsequently, fibrosis [49].

Furthermore, pericardial fat activates the intrinsic autonomic nervous system because some parasympathetic intrinsic ganglia are encased within pericardial fat pads. Increased pericardial fat can locally influence these autonomic ganglia, enhancing vagal tone and increasing the predisposition to AF [50]. Obese people present the most pronounced epicardial adipose-tissue volume and electroanatomical remodeling in the posterior LA, a potentially important substrate for AF in this patient subgroup. Consequently, an ablation strategy that targets posterior-wall isolation in addition to PV isolation could be considered as a targeted therapeutic strategy with satisfactory outcomes in obese patients [51]. In the Framingham Heart Study, which used a middle-aged-to-elderly-community-based cohort, it was observed that larger pericardial fat volumes—as detected by computerized tomography—were associated with a 40% higher likelihood of AF occurrence. This association remained significant and presented consistency even after serial adjustments for clinical AF-risk factors, including BMI, HF, and myocardial infarction. Such a correlation was not observed between AF and intrathoracic or visceral fat [50].

## 6. The Beneficial Role of Weight Loss

Obesity has been proposed as a contributor to the increasing prevalence of AF in Western countries and, thus, weight loss is considered an effective treatment target to reduce AF burden. The LEGACY study demonstrated that progressive weight loss reduced the AF burden in overweight and obese patients with symptomatic AF. Long-term weight loss and weight-goal maintenance led to a six-fold greater freedom from AF. This is explained by the fact that reduced left atrial volume and left ventricular hypertrophy are associated with weight loss. Moreover, weight loss also demonstrated beneficial outcomes for other cardiovascular risk factors and co-morbidities strongly associated with high AF burden, such as diabetes, hypertension, dyslipidemia, and sleep apnea [52].

## 7. Obstructive Sleep Apnea and AF

An underreported cause of atrial fibrillation is obstructive sleep apnea (OSA). Predominantly, male patients are affected. The early recognition of this clinical entity and management with CPAP (continuous positive airway pressure) ameliorates symptoms and apnoeic episodes and, in turn, decreases AF burden. Obesity is strongly correlated with OSA and shares its general pathophysiological mechanisms, as OSA gradually leads to hypertension, heart failure, autonomic imbalance, and inflammation. This is mediated by the direct effect of each apnoeic episode on intrathoracic pressure and oxygen saturation. It is known from the Valsalva-phases physiology that abrupt airway blockage causes increased intrathoracic pressure, which initially increases cardiac output as blood is forced into the left atrium by the compression of pulmonary circulation. However, this is followed by a marked blood-pressure decrease due to impaired venous return to the right cardiac chambers secondary to high intrathoracic pressure. On the other hand, when the apnoiec episode ends and the airway is patent, the intrathoracic pressure normalizes and allows a marked increase in cardiac output, leading to blood-pressure overshooting. At the same time, the episode itself causes hypoxia and hypercapnia, which results in sympathetic stimulation and autonomic dysregulation. Significant fluctuations in preload and afterload are linked to the aforementioned abrupt cardiac-output shift. The constellation of all these factors encourages arterial hypertension and increased left-atrial-wall stress, oxidative stress, and inflammation [53].

## 8. Alcohol and Atrial Fibrillation

Modest alcohol consumption is considered to have a beneficial effect, mainly due to its antioxidant properties. However, excessive consumption can have both an acute (described as “holiday heart syndrome”) and a chronic effect on AF incidence.

Interestingly, AF is not strictly temporally related to the alcohol intoxication phase, but can also occur later at the hangover period. Excessive drinking undoubtedly activates the sympathetic cascade, including increases in heart rate and decreases in heart-rate variability. Alcohol, as a potent AF trigger, exerts a toxic effect on myocardial cell integrity because it causes acute cell injury and oxidative stress. The interplay between sympathetic overdrive and cellular damage leads to altered electrophysiological properties, such as the shortening of the atrial refractory period—in particularly in the pulmonary veins—and decreased intra-atrial conduction time. Both mechanisms facilitate a reentry model that makes AF sustainable.

As stated above, during the hangover period, the individual is dehydrated as alcohol has a marked diuretic effect. Antidiuretic hormones and aldosterone are released to offset alcohol’s action, but the whole process results in an electrolyte imbalance and encourages tachyarrhythmia [54]

Moreover, chronic alcohol consumption drives all the detrimental mechanisms that also lead to a more sustainable and not easily reversible model of AF. Habitual alcohol consumption gradually creates the ideal milieu for AF occurrence. Alcohol intake results in obesity, sleep apnea, and hypertension. Additionally, through cellular and structural mechanisms, alcohol increases oxidative stress, left atrial stretch and, consequently, fibrosis [54].

### Genetics of AF

Heredity has been demonstrated as a common pathogenetic factor in AF. It is now evident that genetic variants are associated with a 40% increased risk of AF [55]. Given the initial identification of a mutation in KCNQ1 that affects potassium channels, many of the ion-channel genes involved in the cardiac action potential and a cascade of mutations have been identified as AF-causing mutations. For instance, KCNQ1 mutations lead to a gain-of-function in the I_Ks_ channel complex, which increases the repolarizing K^+^ current, shortens the action-potential duration (APD) and the atrial refractory period and, in turn, renders the cells susceptible to depolarization by subsequent AF triggers in the form of electrical impulses. Similarly, mutations identified in KCNE1, KCNE2, KCNE3, and KCNE5 have been shown to exert gain-of-function effects on I_Ks_. The other major repolarizing current in the myocardial cells is the rapidly repolarizing potassium current I_Kr_, encoded by KCNH2 and also associated with gain-of-function in I_Ks_.

On the other hand, several reports have described loss-of-function mutations identified in individuals with early-onset lone AF. This implies that the prolongation of the effective refractory period and the action-potential duration can also cause AF. Loss-of-function mutations delay repolarization and encourage Ca^2+^-mediated afterdepolarization, which can, in turn, trigger AF [56]. Such alterations in action-potential duration by either gain- or loss-of-function mutations explain why QTc changes are associated with an increased risk of AF [57].

It was found that sodium-channel-subunit mutations are important factors in the development of familial AF. Six variations in Na^+^-channel genes have been identified: SCN1B, SCN2B, SCN3B, SCN4B, SCN5A, and SCN10A. Both loss-of-function and gain-of-function variations predispose individuals to the formation of an appropriate AF substrate. Gain-of-function mutations may increase AF vulnerability by increasing cellular hyperexcitability, whereas loss-of-function mutations shorten ERP and promote atrial slow-conduction velocity [58,59,60].

However not only gene mutations linked to ion channels account for AF. The GATA4, GATA5, and GATA6 genes are involved in cardiogenesis by encoding transcription factors. Mutations in these genes are related to increased focal activity in the pulmonary veins and abnormal sleeve development. These factors can predispose patients to AF because of increased automaticity and microreentry-circuit formation, respectively. [61,62].

Furthermore, several genes play a crucial role in embryogenesis, such as Nkx2.5 and PITX2. A Nkx2.5-loss-of-function mutation potentially predisposes a patient to the formation of an arrhythmogenic pulmonary sleeve [63,64]. The PITX2 gene takes part in the symmetrical right–left development of the heart, the suppression of sinus-node formation in the left atrium, and the formation of the pulmonary-vein myocardial sleeve [65]. Recent genome-wide association studies reported several risk variants on chromosome 4q25, adjacent to the PITX2 gene, which are associated with AF [66].

To summarize, it is apparent that several pathophysiological mechanisms are responsible for the genesis of AF. Several risk factors, comorbidities, and genes can result in the activation of the pathophysiological cascades mentioned so far and lead to AF-trigger activation and AF perpetuation.

However, apart from the chronic diseases that provoke AF genesis, other reversible causes might also be involved. The most common are AF after myocardial infarction (MI), post-PFO-implant AF, and AF after cardiac surgery, which are analyzed further below.

## 9. Post-MI Atrial Fibrillation

Atrial fibrillation is a common complication of AMI, and it is potentially attributable to causes such as excess circulating catecholamines, left atrial ischemia, significant left ventricular dysfunction, electrolyte disorders, and pericardial inflammation [67]. The REGARDS study, which included 1631 participants with AF and 22,297 participants without AF, showed that AF leads to a twofold increase in the risk of MI during a median follow-up of 4.5 years [68]. The incidence of AF in patients admitted to hospital with AMI and thrombolysis varied between 6.8% and 21% [69]. In Goldberg et al.’s study, 2596 patients with an initial AMI and no previous AF history were included. The incidence of AF post-MI was reduced over the years between 1990 and 1997, from 18% to 11%. This reduction was attributed to the more widespread use of thrombolysis [70]. The TIGRIS Registry enrolled 8277 patients 1 to 3 years post-MI. Of these patients, 8.5% had previous AF, whereas 3% developed AF during a 2-year follow-up. The participants with comorbid AF and prior MI were at a higher risk of thromboembolism and bleeding [71]. Older age, female sex, diabetes mellitus, hypertension, and comorbidities, including chronic kidney disease, were associated with new-onset AF at or after MI. The anterior location of the MI, acute heart failure, and deteriorated left-ventricle contractility were the MI factors associated with newly diagnosed AF at or after MI [72]. In the large prospective TRIUMPH registry of patients with AMI, each twofold increase in NT-proBNP and hs-CRP was associated with 18% and 15% increases in AF incidence, respectively [73].

Inflammation is a determinant factor associated with AF occurrence in myocardial infraction. Indices of inflammation and myocardial stretch, such as hs-CRP and NT-proBNP, are proportionally related to increased rates of AF. This is not in line with the amount of myocardial necrosis expressed by troponin T [73]. Inflammation leads to increased IL-6 levels, which in turn modulates matrix metallopeptidase 2 (MMP2) expression. This stimulates atrial fibrosis formation and dilatation, leading to an anatomical substrate that is vulnerable to AF [74]. Furthermore, direct atrial ischemia and infraction caused by MI result in fibrotic tissue and the creation of appropriate substrates for AF perpetuation [75]. Another potential mechanism of AF occurrence after MI is atrial refractory period shortening. Consequently, heterogeneous conduction contributes to reentry formation [76]. Furthermore, myocardial ischemia is associated with increased levels of intracellular Ca^+2^, which facilitate increased Na^+^–Ca^+2^ exchange currents and focal ectopic activity, including enhanced automaticity, DADs, and EADs [77].

## 10. PFO and Atrial Fibrillation

Another group of patients who develop AF in the modern era is composed of those undergoing post-PFO closure. Two recent randomized trials demonstrated the superiority of PFO closure over antiplatelet treatment in patients with previous cryptogenic stroke [78,79]. The rates of AF were 4.6% and 6.6% in these trials, respectively. The occurrence of AF was device-dependent, with a risk ratio varying from 2.1 to 14.6 [80]. It is noteworthy that most of these episodes (83%) occurred within the first 45 days post-implant [80]. As expected, the stringent monitoring with implantable loop recorders post-PFO implant included both symptomatic and asymptomatic AF episodes and, consequently, the AF incidence increased to almost 29% [81]. Older age (>60 years), male gender, and left atrial disc diameter >25 mm were independent predictors of AF occurrence [81]. Although the AF rate seems to be substantial, it is promising that the majority of these episodes (59%) resolve within 2 weeks, which underscores the potential existence of a reversible mechanistic effect post-implant associated with local atrial stretch and inflammation. However, the pathophysiological mechanism that accounts for early AF occurrence post-PFO implant has not been entirely delineated.

## 11. Post-Cardiac-Surgery AF

Postoperative atrial fibrillation (POAF), defined as newly diagnosed atrial fibrillation (AF) in the immediate post-surgery period, is the most common and important type of secondary AF. It emerges in around 35% of cardiac surgery patients and is associated with multiple adverse events, such as heart failure, cerebrovascular events, and elevated overall costs [82,83,84,85]. The emergence of POAF after cardiac surgery is associated with increased short-term and long-term morbidity and mortality [86]. The high long-term AF-recurrence rate raises questions as to the completely temporary nature of POAF and suggests an interaction between transient periprocedural factors and a pre-existing substrate. However, the AF-recurrence rate in patients who develop POAF after cardiothoracic surgery (46%) is lower than that in patients with POAF after non-thoracic surgery (64%), which supports the idea that transient factors play a greater role than pre-existing substrates. Moreover, the long-term risk of stroke is lower with POAF after cardiac surgery than with POAF after non-cardiothoracic surgery. The high AF-recurrence rates in patients with POAF make this condition a definite marker of subsequent risk of AF [87]. It is estimated that 20–30% of CABG patients are diagnosed with clinically significant AF that requires medical intervention. Those at highest risk are older male patients with higher numbers of grafts or left-internal-mammary-artery-bypass grafting, the presence of right-coronary-artery disease, and longer aortic cross-clamp [88].

The inflammatory response during surgery plays a major causative role in postoperative AF pathogenesis. Cardiac surgery with cardiopulmonary bypass (CPB) induces systemic inflammatory response syndrome (SIRS). The contact between the blood components and the artificial surface of the bypass circuit, operative trauma, ischemia–reperfusion injury endotoxemia, and complement activation are all potential inflammatory factors [89]. Specifically, CPB contributes to the release of pro-inflammatory interleukin-8 (IL-8) and tumor necrosis factor (TNF), which is offset partially by the anti-inflammatory cytokine IL-10. The levels of IL-6 were demonstrated to be lower when minimally invasive coronary-artery bypass is applied compared to the off-pump technique. This underscores the significance of operative injury, rather than CPB per se. Furthermore, CPB contributes to the activation of neutrophils, according to assessments with plasma elastase levels and bactericidal permeability increasing protein (BPI). This inflammatory reaction may lead to the appearance of postoperative complications, including myocardial dysfunction, neurologic dysfunction, respiratory and renal failure, bleeding disorders, abnormal liver function, and multiple organ failure (MOF). Additionally, CPB and surgical trauma can lead to the production of all these different proinflammatory mediators alongside widespread endothelial activation, with increases in the expression of adhesion molecules and the impaired release of nitric oxide. Increased levels of the anti-inflammatory cytokines, TNF-α and CRP, have been linked with POAF [90].

Oxidative stress plays a major role in post-cardiac-surgery AF as the aftermath of the open-heart-surgery inflammatory response. Oxidative stress is the outcome of the uncontrolled production of reactive oxygen species (ROS), which overwhelm endogenous antioxidant capabilities. Preoperative disease states, such as myocardial ischemia, diabetes, and atherosclerosis, are associated with oxidative stress. Moreover, any potential blood transfusion due to blood loss or damage during CPB contributes to increased oxidative stress. Reactive oxygen species can also be produced by dysregulated vascular endothelial cells and by reperfusion injury. Ischemic injury results in the accumulation of activated neutrophils in the myocardium and, subsequently, the release of ROS and other proteolytic enzymes. Further myocardial damage occurs during reperfusion as a consequence of mitochondrial dysfunction, which is driven by ROS production. Moreover, high ROS levels can modulate multiple signaling pathways and transcription factors, increasing inflammation, apoptosis, or necrosis. Oxidative stress associated with CPB and cardiac arrest has been linked to the disruption of electrical activity and modified atrial remodeling [91]. Studies showed that NADPH-stimulated superoxide generation in the right atrial appendage is independently associated with a higher risk of post-operative AF [92].

Furthermore, the integrity of the pericardial membrane surrounding the heart is disrupted during cardiac surgery. As a result, pericardial fluid contains high concentrations of oxidized hemoglobin, as well as elevated levels of neutrophils and monocytes, which increase oxidative stress within the pericardial space. All these elements lead to electromechanical disturbances, which are the driving forces of AF [93].

Lastly, electrolytic disorders (hypomagnesemia and hypokalemia) are common findings in perioperative and postoperative situations, and they contribute to AF onset. Hypomagnesemia is a common post-surgery laboratory finding and has been identified as a predictor of POAF, as low magnesium levels enhance atrial tissue automaticity. Hypokalemia leads to cellular hyperpolarity, enhanced automaticity, and increased resting potential, which also may be contributors to POAF [94].

## 12. Future Directions

As cases of AF are expected to exponentially increase in the next decades, action has to be taken soon to target AF holistically, beyond routine medical and interventional treatments. As has already elucidated by the known mechanisms, the roles of oxidative stress and atrial fibrosis are of paramount importance. Therefore, a strict approach with the goal of eliminating all the potential risk factors should be implemented. The role of imaging might be more promising in two directions, the preventive and the therapeutic. Novel imaging techniques might offer more data about atrial tissue properties before episodes of AF, stratifying patients that are at higher risk of AF incidence. This can be achieved by non-invasive methods, such as the detection of atrial strain by echocardiography or of atrial fibrosis by MRI scanning, respectively. Moreover, in the future, multielectrode and more sophisticated mapping may make it possible to map AF episodes outside of the laboratory and increase awareness of the triggering mechanisms beforehand. Such information would enable operators performing AF ablation to be more precise and combat AF on an individual and tailored basis.

In conclusion, the mechanisms of atrial fibrillation are complex and multifactorial. All the comorbidities and predisposing factors that are known to be related in our clinical practice seem to drive different pathways, which have two main targets. The first is the activation of triggers in vulnerable regions, such as the pulmonary veins, in most cases. The second is the formation of a favorable substrate that accounts for atrial fibrillation sustainability. Based on the latest knowledge about the mechanisms that increase the activation of triggers and AF perpetuation—along with further improvements in mapping technology—therapeutic strategies should aim to eliminate triggers and modify the atrial substrate.

## Data Availability

No new data were created or analyzed in this study. Data sharing is not applicable to this article.

## References

[1] Heeringa J., van der Kuip D.A., Hofman A., Kors J.A., van Herpen G., Stricker BH C., Stijnen T., Lip G.Y.H., Witteman J.C.M. (2006). Prevalence, incidence and lifetime risk of atrial fibrillation: The Rotterdam study. Eur. Heart J..

[2] Zoni-Berisso M., Lercari F., Carazza T., Domenicucci S. (2014). Epidemiology of atrial fibrillation: European perspective. Clin. Epidemiol..

[3] Kornej J., Börschel C.S., Benjamin E.J., Schnabel R.B. (2020). Epidemiology of Atrial Fibrillation in the 21st CenturyNovel Methods and New Insights. Circ. Res..

[4] Tsigkas G., Apostolos A., Despotopoulos S., Vasilagkos G., Kallergis E., Leventopoulos G., Mplani V., Davlouros P. (2022). Heart failure and atrial fibrillation: New concepts in pathophysiology, management, and future directions. Heart Fail. Rev..

[5] Olshansky B., Goldberger Z.D., Pogwizd S.M., Knight B.P., Yeon S.B., FACC, FHRS (2021). The electrocardiogram in atrial. UpToDate.

[6] Steinberg J.S., O’Connell H., Li S., Ziegler P.D. (2018). Thirty-second gold standard definition of atrial fibrillation and its relationship with subsequent arrhythmia patterns: Analysis of a large prospective device database. Circ. Arrhythmia Electrophysiol..

[7] Hindricks G., Potpara T., Dagres N., Arbelo E., Bax J.J., Blomström-Lundqvist C., Boriani G., Castella M., Dan G.-A., Dilaveris P.E. (2020). ESC Scientific Document Group 2020 ESC Guidelines for the diagnosis and management of atrial fibrillation developed in collaboration with the European Association for Cardio-Thoracic Surgery (EACTS). Eur. Heart J..

[8] Mackstaller L.L., Alpert J.S. (1997). Atrial Fibrillation: A Review of Mechanism, Etiology, and Therapy. Clin. Cardiol..

[9] Haissaguerre M., Jaïs P., Shah D.C., Takahashi A., Hocini M., Quiniou G., Garrigue S., Mouroux A.L., Métayer P.L., Clémenty J. (1998). Spontaneous Initiation of Atrial Fibrillation by Ectopic Beats Originating in the Pulmonary Veins. N. Engl. J. Med..

[10] Nattel S. (2003). Atrial Electrophysiology and Mechanisms of Atrial Fibrillation. J. Cardiovasc. Pharmacol. Ther..

[11] Ehrlich J.R., Cha T.J., Zhang L., Chartier D., Melnyk P., Hohnloser S.H., Nattel S. (2003). Cellular electrophysiology of canine pulmonary vein cardiomyocytes: Action potential and ionic current properties. J. Physiol..

[12] Weber K.T., Brilla C.G., Campbell S.E., Guarda E., Zhou G., Sriram K. (1993). Myocardial fibrosis: Role of angiotensin II and aldosterone. Angiotensin Heart.

[13] Harada M., Van Wagoner D.R., Nattel S. (2015). Role of Inflammation in Atrial Fibrillation Pathophysiology and Management. Circ. J..

[14] Voigt N., Dobrev D. (2013). The biology of human pulmonary veins: Does it help us to better understand AF pathophysiology in patients?. Heart Rhythm.

[15] Santangeli P., Marchlinski F.E. (2017). Techniques for the provocation, localization, and ablation of non–pulmonary vein triggers for atrial fibrillation. Heart Rhythm.

[16] Nagarakanti R., Ung K., Strahan H. (2018). Critical Role of the Posterior Left Atrium in the Perpetuation of Persistent Atrial Fibrillation and the Hybrid Ablation Approach for Persistent Atrial Fibrillation Management: A Single-center Outcomes Study. J. Innov. Card. Rhythm Manag..

[17] Kistler P.M., Chieng D., Sugumar H., Ling L.H., Segan L., Azzopardi S., Al-Kaisey A., Parameswaran R., Anderson R.D., Hawson J. (2023). Effect of Catheter Ablation Using Pulmonary Vein Isolation With vs Without Posterior Left Atrial Wall Isolation on Atrial Arrhythmia Recurrence in Patients With Persistent Atrial Fibrillation The CAPLA Randomized Clinical Trial. JAMA.

[18] Sauer W.H., Alonso C., Zado E., Cooper J.M., Lin D., Dixit S., Russo A., Verdino R., Ji S., Gerstenfeld E.P. (2006). Atrioventricular nodal reentrant tachycardia in patients referred for atrial fibrillation ablation: Response to ablation that incorporates slow-pathway modification. Circulation.

[19] Moe G.K., Abildskov J.A. (1959). Atrial fibrillation as a self-sustaining arrhythmia independent of focal discharge. Am. Heart J..

[20] Allessie M.A., de Groot N.M., Houben R.P., Schotten U., Boersma E., Smeets J.L., Crijns H.J. (2010). Electropathological substrate of long-standing persistent atrial fibrillation in patients with structural heart disease: Longitudinal dissociation. Circ. Arrhythmia Electrophysiol..

[21] Jalife J., Berenfeld O., Mansour M. (2002). Mother rotors and fibrillatory conduction: A mechanism of atrial fibrillation. Cardiovasc. Res..

[22] Aronis K.N., Berger R.D., Ashikaga H. (2017). Rotors How Do We Know When They Are Real?. Circ. Arrhythmia Electrophysiol..

[23] Narayan S.M., Krummen D.E., Shivkumar K., Clopton P., Rappel W.J., Miller J.M. (2012). Treatment of Atrial Fibrillation by the Ablation of Localized Sources CONFIRM (Conventional Ablation for Atrial Fibrillation With or Without Focal Impulse and Rotor Modulation). J. Am. Coll. Cardiol..

[24] Parameswaran R., Voskoboinik A., Gorelik A., Lee G., Kistler P.M., Sanders P., Kalman J.M. (2018). Clinical impact of rotor ablation in atrial fibrillation: A systematic review. Europace.

[25] Cuculich P.S., Wang Y., Lindsay B.D., Faddis M.N., Schuessler R.B., Damiano R.J., Li L., Rudy Y. (2010). Non invasive characterization of epicardial activation in humans with diverse atrial fibrillation patterns. Circulation.

[26] Ausma J., Wijffels M., Thoné F., Wouters L., Allessie M., Borgers M. (1997). Structural changes of atrial myocardium due to sustained atrial fibrillation in the goat. Circulation.

[27] Frustaci A., Chimenti C., Bellocci F., Morgante E., Russo M.A., Maseri A. (1997). Histological substrate of atrial biopsies in patients with lone atrial fibrillation. Circulation.

[28] Wijesurendra R.S., Casadei B. (2019). Mechanisms of atrial fibrillation. Heart.

[29] Tahhan A.S. (2017). Association between oxidative stress and atrial fibrillation. Heart Rhythm.

[30] Rahmutula D., Marcus G.M., Wilson E.E., Ding C.H., Xiao Y., Paquet A.C. (2013). Molecular basis of selective atrial fibrosis due to overexpression of transforming growth factor-beta1. Cardiovasc. Res..

[31] Tan A.Y., Zimetbaum P. (2011). Atrial fibrillation and atrial fibrosis. Cardiovasc. Pharmacol..

[32] Harada M., Luo X., Qi X.Y., Tadevosyan A., Maguy A., Ordog B., Ledoux J., Kato T., Naud P., Voigt N. (2012). Transient Receptor Potential Canonical-3 Channel–Dependent Fibroblast Regulation in Atrial Fibrillation. Circulation.

[33] Yoo S., Aistrup G., Shiferaw Y., Ng J., Mohler P.J., Hund T.J., Waugh T., Browne S., Gussak G., Gilani M. (2018). Oxidative stress creates a unique CaMKII-mediated substrate for atrial fibrillation in heart failure. JCI Insight.

[34] Ho E., Galougahi K.K., Liu C.C., Bhindi R., Figtree G.A. (2013). Biological markers of oxidative stress: Applications to cardiovascular research practice. Redox Biol..

[35] Sagris M., Vardas E.P., Theofilis P., Antonopoulos A.S., Oikonomou E., Tousoulis D. (2022). Antonopoulos, Evangelos Oikonomou and Dimitris Tousoulis Atrial Fibrillation: Pathogenesis, Predisposing Factors, and Genetics. Int. J. Mol. Sci..

[36] Zaidi Y., Aguilar E.G., Troncoso M., Ilatovskaya D.V., DeLeon-Pennell K.Y. (2021). Immune regulation of cardiac fibrosis post myocardial infarction. Cell Signal..

[37] Dumitriu I.E., Dimou P., Kaur S., Dinkla S., Kaski J.C., Camm A.J. (2020). Increase in inflammatory T cell subsets in atrial fibrillation: The missing link underlying inflammation in AF. Eur. Heart J..

[38] Legere S.A., Haidl I.D., Légaré J.F., Marshall J.S. (2019). Mast Cells in Cardiac Fibrosis: New Insights Suggest Opportunities for Intervention. Front. Immunol..

[39] Murray D.B., McLarty-Williams J., Nagalla K.T., Janicki J.S. (2012). Tryptase activates isolated adult cardiac fibroblasts via protease activated receptor-2 (PAR-2). J. Cell Commun. Signal..

[40] Shiota N., Jin D., Takai S., Kawamura T., Koyama M., Nakamura N. (1997). Miyazaki Chymase is activated in the hamster heart following ventricular fibrosis during the chronic stage of hypertension. FEBS Lett..

[41] Deb B., Ganesan P., Feng R., Narayan S.M. (2021). Identifying Atrial Fibrillation Mechanisms for Personalized Medicine. J. Clin. Med..

[42] Iwasaki Y.K., Nishida K., Kato T., Nattel S. (2011). Atrial Fibrillation Pathophysiology Implications for Management. Circulation.

[43] Heijman J., Voigt N., Nattel S., Dobrev D. (2014). Cellular and molecular electrophysiology of atrial fibrillation initiation, maintenance, and progression. Circ. Res..

[44] Chaldoupi S.M., Loh P., Hauer R.N., De Bakker J.M., van Rijen H.V. (2009). The role of connexin40 in atrial fibrillation. Cardiovasc. Res..

[45] Marrouche N.F., Wilber D., Hindricks G., Jais P., Akoum N., Marchlinski F., Kholmovski E., Burgon N., Hu N., Mont L. (2014). Association of Atrial Tissue Fibrosis Identified by Delayed Enhancement MRI and Atrial Fibrillation Catheter Ablation The DECAAF Study. JAMA.

[46] Marrouche N.F., Greene T., Dean J.M., Kholmovski E.G., Boer LM D., Mansour M., Calkins H., Marchlinski F., Wilber D., Hindricks G. (2021). Efficacy of LGE-MRI-guided fibrosis ablation versus conventional catheter ablation of atrial fibrillation: The DECAAF II trial: Study design. J. Cardiovasc. Electrophysiol..

[47] Chen P.S., Chen L.S., Fishbein M.C., Lin S.F., Nattel S. (2014). Role of the Autonomic Nervous System in Atrial Fibrillation Pathophysiology and Therapy. Circ. Res..

[48] Lau D.H., Schotten U., Mahajan R., Antic N.A., Hatem S.N., Pathak R.K., Hendriks J.M.L., Kalman J.M., Sanders P. (2016). Novel mechanisms in the pathogenesis of atrial fibrillation: Practical applications. Eur. Heart J..

[49] Stephane N. (2014). Hatem and Prashanthan Sanders Epicardial adipose tissue and atrial fibrillation. Cardiovasc. Res..

[50] Thanassoulis G., Massaro J.M., O’Donnell C.J., Hoffmann U., Levy D., Ellinor P.T., Wang T.J., Schnabel R.B., Vasan R.S., Fox C.S. (2010). Pericardial fat is associated with prevalent atrial fibrillation: The Framingham Heart Study. Circ. Arrhythmia Electrophysiol..

[51] Mahajan R., Nelson A., Pathak R.K., Middeldorp M.E., Wong C.X., Twomey D.J., Carbone A., Teo K., Agbaedeng T., Linz D. (2018). Electroanatomical Remodeling of the Atria in Obesity: Impact of Adjacent Epicardial Fat. JACC Clin. Electrophysiol..

[52] Pathak R.K., Middeldorp M.E., Meredith M., Mehta A.B., Mahajan R., Wong C.X. (2015). Long-Term Effect of Goal-Directed Weight Management in an Atrial Fibrillation CohortA Long-Term Follow-Up Study (LEGACY). J. Am. Coll. Cardiol..

[53] Yeghiazarians Y., Jneid H., Tietjens J.R., Redline S., Brown D.L., El-Sherif N., Mehra R., Bozkurt B., Ndumele C.E., Somers V.K. (2021). Obstructive Sleep Apnea and Cardiovascular Disease: A Scientific Statement From the American Heart Association. Circulation.

[54] Voskoboinik A., Prabhu S., Ling L.H., Kalman J.M., Kistler P.M. (2016). Alcohol and Atrial Fibrillation. J. Am. Coll. Cardiol..

[55] Lubitz S.A., Yin X., Fontes J.D., Magnani J.W., Rienstra M., Pai M. (2010). Association Between Familial Atrial Fibrillation and Risk of New-Onset Atrial Fibrillation. JAMA.

[56] Christophersen I.E., Ellinor P.T. (2016). Genetics of atrial fibrillation: From families to genomes. J. Hum. Genet..

[57] Nielsen J.B., Graff C., Pietersen A., Lind B., Struijk J.J., Olesen M.S., Haunsø S., Gerds T.A., Svendsen J.H., Køber L. (2013). J-shaped association between QTc interval duration and the risk of atrial fibrillation: Results from the Copenhagen ECG Study. J. Am. Coll. Cardiol..

[58] Tucker N.R., Ellinor P.T. (2014). Ellinor Emerging Directions in the Genetics of Atrial Fibrillation. Circ. Res..

[59] Li Q., Huang H., Liu G., Lam K., Rutberg J., Green M.S. (2009). Gain-of-function mutation of Na_v_1.5 in atrial fibrillation enhances cellular excitability and lowers the threshold for action potential firing. Biochem. Biophys. Res. Commun..

[60] Yang Y., Wang M., Zhang X., Tan H.W., Shi H.F., Jiang W.F., Wang X.H., Gang W.Y. (2011). GATA4 loss-of-function mutations in familial atrial fibrillation. Clin. Chim. Acta.

[61] Yang Y.Q., Wang J., Wang X.H., Wang Q., Tan H.W., Zhang M., Shen F.F., Jiang J.Q., Fang W.Y., Liu X. (2012). Mutational spectrum of the GATA5 gene associated with familial atrial fibrillation. Int. J. Cardiol..

[62] Yang Y., Wang X., Tan H.W., Jiang W.F., Fang W.Y., Liu X. (2012). Prevalence and spectrum of GATA6 mutations associated with familial atrial fibrillation. Int. J. Cardiol..

[63] Xie W.H., Chang C., Xu Y.J., Li R.G., Qu X.K., Fang W.Y., Liu X., Yang Y.Q. (2013). Prevalence and spectrum of Nkx2.5 mutations associated with idiopathic atrial fibrillation. Clinics.

[64] Wang J., Zhang D.F., Sun Y.M., Li R.G., Qui X.B., Qu X.K., Liu X., Gang W.Y., Yang Y.Q. (2014). NKX2-6 mutation predisposes to familial atrial fibrillation. Int. J. Mol. Med..

[65] Feghaly J., Zakka P., London B., MacRae C.A., Refaat M.M. (2018). Genetics of Atrial. J. Am. Heart Assoc..

[66] Chinchilla A., Daimi H., Lozano-Velasco E., Dominguez J.N., Caballero R., Delpón E. (2011). *PITX2* Insufficiency Leads to Atrial Electrical and Structural Remodeling Linked to Arrhythmogenesis. Circ. Cardiovasc. Genet..

[67] Bhatia G.S., Lip G.Y. (2004). Atrial Fibrillation Post-Myocardial Infarction: Frequency, Consequences and Management. Curr. Heart Fail. Rep..

[68] Soliman E.Z., Safford M.M., Muntner P., Khodneva Y., Dawood F.Z., Zakai N.A., Thacker E.L., Judd S., Howard V.J., Howard G. (2014). Atrial fibrillation and the risk of myocardial infraction. JAMA Intern. Med..

[69] Schmitt J., Duray G., Gersh B.J., Hohnloser S.H. (2009). Atrial fibrillation in acute myocardial infarction: A systematic review of the incidence, clinical features and prognostic implications. Eur. Heart J..

[70] Goldberg R.J., Yarzebski J., Lessard D., Wu J., Gore J.M. (2002). Recent trends in the incidence rates of and death rates from atrial fibrillation complicating initial acute myocardial infarction: A community-wide perspective. Am. Heart J..

[71] Carnicelli A.P., Owen R., Pocock S.J., Brieger D.B., Yasuda S., Nicolau J.C. (2021). Atrial fibrillation and clinical outcomes 1 to 3 years after myocardial infarction. Open Heart.

[72] Jabre P., Jouven X., Adnet F., Thabut G., Bielinski S.J., Weston S.A., Roger V.L. (2011). Atrial Fibrillation and Death After Myocardial. Circulation.

[73] Parashar S., Kella D., Reid K.J., Spertus J.A., Tang F., Langberg J., Vaccarino V., Kontos M.C., Lopes R.D., Lloyd M.S. (2013). New-Onset Atrial Fibrillation After Acute Myocardial Infarction and Its Relation to Admission Biomarkers (from the TRIUMPH Registry) Susmita. Am. J. Cardiol..

[74] Xu Y., Sharma D., Du F., Liu Y. (2013). The role of Toll-like receptor 2 and hypoxia-induced transcription factor-1α in the atrial structural remodeling of non-valvular atrial fibrillation. Int. J. Cardiol..

[75] Liang F., Wang Y. (2021). Coronary heart disease and atrial fibrillation: A vicious cycle. Am. J. Physiol. Heart Circ. Physiol..

[76] Zukela T., Zhou Q., Wang H., Zhou X., Li Y., Zhang Y. (2015). Relationship between new-onset atrial fibrillation and sympathetic neural remodeling in a canine acute myocardial infarction model. Zhonghua Xin Xue Guan Bing Za Zhi.

[77] Landstrom A.P., Dobrev D., Wehrens X.H. (2017). Calcium Signaling and Cardiac Arrhythmias. Circ. Res..

[78] Mas J.L., Derumeaux G., Guillon B., Massardier E., Hosseini H., Mechtouff L., Arquizan C., Béjot Y., Vuillier F., Detante O. (2017). Patent foramen ovale closure or anticoagulation vs. antiplatelets after stroke. N. Engl. J. Med..

[79] Søndergaard L., Kasner S.E., Rhodes J.F., Andersen G., Iversen H.K., Nielsen-Kudsk J.E., Settergren M., Sjöstrand C., Roine R.O., Hildick-Smith D. (2017). Patent foramen ovale closure or antiplatelet therapy for cryptogenic stroke. N. Engl. J. Med..

[80] Mojadidi M.K., Zaman M.O., Elgendy I.Y., Mahmoud A.N., Patel N.K., Agarwal N., Tobis J.M., Meier B. (2018). Cryptogenic Stroke and Patent Foramen Ovale. J. Am. Coll. Cardiol..

[81] Guedeney P., Laredo M., Zeitouni M., Hauguel-Moreau M., Wallet T., Elegamandji B., Alamowitch S., Crozier S., Sabben C., Deltour S. (2022). Supraventricular Arrhythmia Following Patent Foramen Ovale Percutaneous Closure. Cardiovasc. Interv..

[82] Frendl G., Sodickson A.C., Chung M.K., Waldo A.L., Gersh B.J., Tisdale J.E., Calkins H., Aranki S., Kaneko T., Cassivi S. (2014). AATS guidelines for the prevention and management of perioperative atrial fibrillation and flutter for thoracic surgical procedures. J. Thorac. Cardiovasc. Surg..

[83] Philip I., Berroeta C., Leblanc I. (2014). Perioperative challenges of atrial fibrillation. Curr. Opin. Anesthesiol..

[84] Lubitz S.A., Yin X., Rienstra M., Schnabel R.B., Walkey A.J., Magnani J.W., Rahman F., McManus D.D., Tadros T.M., Levy D. (2015). Long-term outcomes of secondary atrial fibrillation in the community: The Framingham Heart Study. Circulation.

[85] Villareal R.P., Hariharan R., Liu B.C., Kar B., Lee V.V., Elayda M., Lopez J.A., Rasekh A., Wilson J.M., Massumi A. (2004). Postoperative atrial fibrillation and mortality after coronary artery bypass surgery. J. Am. Coll. Cardiol..

[86] Wang M.K., Meyre P.B., Heo R., Devereaux P.J., Birchenough L., Whitlock R., McIntyre W.F., Chen Y.C.P., Ali M.Z., Biancari F. (2022). Short-term and Long-term Risk of Stroke in Patients With Perioperative Atrial Fibrillation After Cardiac Surgery: Systematic Review and Meta-analysis. CJC Open.

[87] Olshansky B. (1996). Management of Atrial Fibrillation After Coronary Artery Bypass Graft. Am. J. Cardiol..

[88] Dobrev D., Aguilar M., Heijman J., Guichard J.B., Nattel S. (2019). Postoperative atrial fibrillation: Mechanisms, manifestations and management. Nat. Rev. Cardiol..

[89] Paparella D., Yau T.M., Young E. (2002). Cardiopulmonary bypass induced inflammation:pathophysiology and treatment. An update. Eur. J. Cardio Thorac. Surg..

[90] Asimakopoulos G. (2001). Systemic inflammation and cardiac surgery: An update. Perfusion.

[91] Zakkar M., Ascione R., James A.F., Angelini G.D., Suleiman M.S. (2015). Inflammation, oxidative stress and postoperative atrial fibrillation in cardiac surgery. Pharmacol. Ther..

[92] Kim Y.M., Kattach H., Ratnatunga C., Pillai R., Channon K.M., Casadei B. (2008). Association of Atrial Nicotinamide Adenine Dinucleotide Phosphate Oxidase Activity With the Development of Atrial Fibrillation After Cardiac Surgery. J. Am. Coll. Cardiol..

[93] Kramer P.A., Chacko B.K., Ravi S., Johnson M.S., Mitchell T., Barnes S., Arabshahi A., Dell’Italia L.J., George D.J., Steele C. (2015). Hemoglobin-associated oxidative stress in the pericardial compartment of postoperative cardiac surgery patients. Lab. Investig..

[94] Greenberg J.W., Lancaster T.S., Schuessler R.B., Melby S.J. (2017). Postoperative atrial fibrillation following cardiac surgery: A persistent complication. Eur. J. Cardio-Thorac. Surg..

